# A Narrative Review of Circular RNAs in Brain Development and Diseases of Preterm Infants

**DOI:** 10.3389/fped.2021.706012

**Published:** 2021-09-21

**Authors:** Qianying Gu, Heng Liu, Jingjing Ma, Jiaming Yuan, Xinger Li, Lixing Qiao

**Affiliations:** ^1^School of Medicine, Southeast University, Nanjing, China; ^2^Department of Pediatrics, Zhongda Hospital, Southeast University, Nanjing, China; ^3^Department of Pediatrics, Tianchang People's Hospital, Anhui, China; ^4^Department of Biobank, Zhongda Hospital, Southeast University, Nanjing, China

**Keywords:** circular RNA, preterm infants, neurological impairment, white matter damage, hypoxic-ischemic encephalopathy

## Abstract

Circular RNAs (circRNAs) generated by back-splicing are the vital class of non-coding RNAs (ncRNAs). Circular RNAs are highly abundant and stable in eukaryotes, and many of them are evolutionarily conserved. They are blessed with higher expression in mammalian brains and could take part in the regulation of physiological and pathophysiological processes. In addition, premature birth is important in neurodevelopmental diseases. Brain damage in preterm infants may represent the main cause of long-term neurodevelopmental disorders in surviving babies. Until recently, more and more researches have been evidenced that circRNAs are involved in the pathogenesis of encephalopathy of premature. We aim at explaining neuroinflammation promoting the brain damage. In this review, we summarize the current findings of circRNAs properties, expression, and functions, as well as their significances in the neurodevelopmental impairments, white matter damage (WMD) and hypoxic-ischemic encephalopathy (HIE). So we think that circRNAs have a direct impact on neurodevelopment and brain injury, and will be a powerful tool in the repair of the injured immature brain. Even though their exact roles and mechanisms of gene regulation remain elusive, circRNAs have potential applications as diagnostic biomarkers for brain damage and the target for neuroprotective intervention.

## Introduction

The damage throughout perinatal period has a profound impact on brain development which is rather essential. Compared with term infants, brain damage in preterm infants, especially the very low birth weight (VLBW) infants, tends to represent the main cause of higher mortality and long-term adverse neurodevelopmental manifestations, including but not limited to motor and sensory problems, decreased cognitive development, and behavior disorders (1, 2). Generally speaking, the lower the gestational age (GA) and birth weight (BW) are, alone or combined, the higher the morbidity of brain damage is, and so is the mortality (3). A previous study has shown that premature birth does play an important role (4). Prematurity may greatly affect the integrity of brain structure and function, as well as health and development of further life (5). Thus, brain damage in preterm infants has grown into a crucial public health issue worldwide. Fortunately, early interventions could be helpful for regeneration and repair of the premature brain.

As is known to all, circular RNAs (circRNAs) have been confirmed to serve as important roles in the pathogenesis of various diseases (6). Currently, researchers have mostly studied circRNAs, and they demonstrated that circRNAs possibility participate in central nervous system (CNS) development and brain damage via excitotoxicity, apoptosis, especially the excessive neuroinflammation (7, 8). For example, circPTK2/miR-29b-SOCS-1-JAK2/STAT3-IL-1β axis could modulate microglia-induced neuronal apoptosis (9). Even though the biological importance of circRNAs has been shown, we know little about expression patterns and biological functions during brain injury in babies who are born prematurely. As a consequence, we mainly summarize the progress achieved in this research field to have a better understanding of the molecular etiology in brain development and damage of preterm infants, and then contribute circRNAs as novel agents for early detection, early diagnosis, early treatment, and even prognosis of circRNA-related diseases in the CNS.

## Characteristics of circRNAs

### Structure

**Figure 1:**
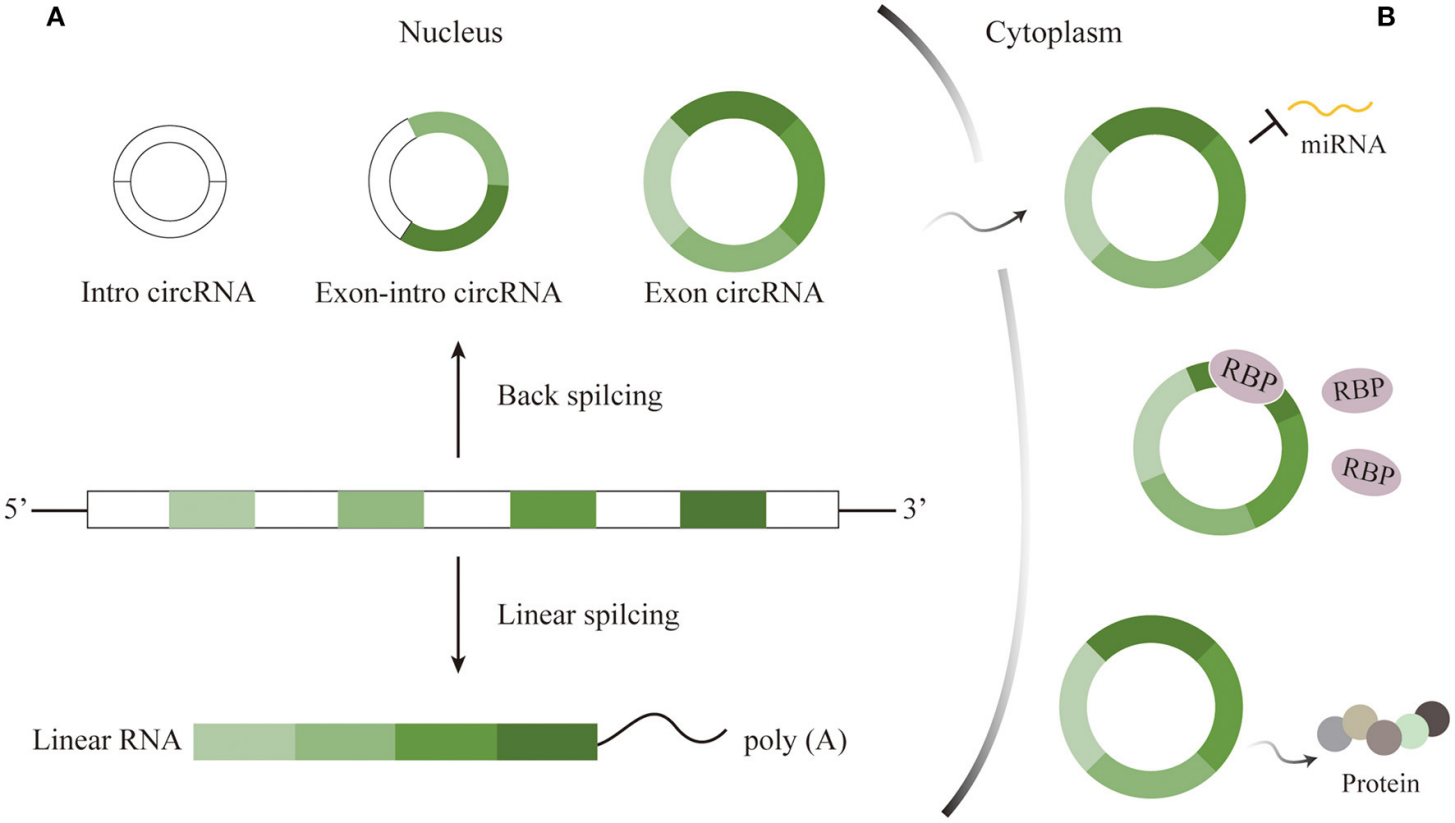
Characteristics and functions of circRNAs. **(A)** Characteristics of circRNAs. circRNAs generated through back splicing can be divided into intron circRNAs, exon-intron circRNAs, and exon circRNAs. The first two kinds remain in nucleus, while exon circRNAs then localize to cytoplasm to play roles. **(B)** Functions of circRNAs. CircRNAs could serve as miRNA sponges, interact with RBP, and translate functional proteins to take part in important biological activities.

Circular RNAs, one class of non-coding RNAs (ncRNAs), can be mainly divided into three categories, containing intron circRNAs, exon-intron circRNAs, and exon circRNAs (10). The first two subtypes remain in nucleus and could alter primary genes, while the last, the predominant one, then localize and function in the cytoplasm after being transferred from the nucleus through a length-dependent pathway (11, 12). Back splicing and linear splicing could independently produce circRNAs and mRNAs. Most circRNAs are generated from protein-coding genes by the reverse connection of the downstream 5′ splice site with the upstream 3′ splice site. Via the specific splicing, circRNAs are single-strained and blessed with covalently closed-loop structure for lack of 5′ and 3′ ends and poly(A) tail whose appearance is totally different from corresponding linear RNAs (Figure 1A) (13, 14).

### Stability

In spite that the production of circRNAs is not efficient in a high rate any more, they are rather resistant to degradation mediated by ribonuclease R (RNase R) for lack of free terminals. As a result, this class of RNAs owns transcript half-life exceeding 48 h longer than most of linear RNAs in cells (15, 16). For example, certain circRNA has six times resistance as many as that of linear transcript after RNase R disposition (17). On the one hand, it is the unique characteristic that renders circRNAs extraordinary stable, which makes it possible for circRNAs to impress the information efficiently and to be ideal diagnostic biomarkers for certain diseases. On the other hand, circRNAs generally accumulate in cytoplasm for free of degeneration, indicating that circRNAs may function in regulation of cellular functions and gene expression.

### Conservation

A majority of circRNAs are evolutionarily conserved in mammals (18, 19). The conservation of circRNAs, at least to a certain extent, is correlated with the complementary sequences in introns flanking exons in both species, instead of the circRNA sequences themselves (20, 21). On this account, the conserved expression of circRNAs in mammals suggests the similarity of their biological process in biogenesis, rather than side products of precursor RNAs.

## CircRNAs in Normal Condition of the Nerve System

### Expression and Distribution

Circular RNAs are widespread found in all kingdoms of life. Their expression is validated in RNA samples from almost all of the organs and tissues, such as brain, testis, kidney, spleen, liver, heart, bone marrow, smooth, skeletal muscles, and so on (22–24). Owing to better stability, these small molecules wrapped in exosomes gain the ability to cross the blood-brain barrier (BBB) and then enter the circulatory system with their original patterns in those of brain (25). Some scientists found 104,388, 96,675, and 82,321 circRNAs from humans, macaques, and mice, respectively, and the average proportion of each species successfully assembled into full-length transcripts was 72.6% (23). This large-scale study also confirmed that circRNAs were tissue-specific and appropriately 20% were expressed in the brain. That is to say, circRNAs are more significantly abundant, dynamically expressed in the mammalian brain tissue than in any other organs (26, 27). This discovery may demonstrate potential functions during nervous system development.

Generally speaking, circRNAs overall transcript levels in brain steadily grow during the developmental and aging phases, from embryonic to adulthood (28, 29). Circular RNAs expression during four different developmental stages (2, 6, 21, and 104 weeks) in rat brain tissues was previously studied, showing an increase in abundance as rats aged (30). However, the reality is that the expression and distribution of certain circRNAs are likely to vary from development to development (31). Some circRNAs are downregulated while the others are in upregulation with maturation (32). In general, this phenomenon in detail remains unclear.

What's more, the expression of circRNAs is not equally distributed throughout the brain, accompanying significant differences in various brain regions (11). Interestingly, circRNAs are highly enriched in olfactory bulb, prefrontal cortex, hippocampus, and cerebellum (33). Besides, the transcriptions of genes that major in producing circRNAs upon the differentiation of complex morphological neurons are dramatically enhanced, indicating that highly differentiated cells contain significantly higher levels of circRNAs (34–36). And circRNAs are unevenly expressed in diverse compartment of neurons, such as cell bodies and axons, but highly localized in synapses or other term. The levels of circRNAs in synapsis are modulated by neuronal activity and plasticity (37). All in all, circRNAs in neurons possess the characteristic of functional diversity as likely as not.

As stated above, circRNAs are dramatically enriched in CNS due to the following reasons. (A) CircRNAs are produced mainly from exons of protein-coding gene which are specific for neuronal and synaptic functions. (B) CircRNAs are unable to be identified by RNase R and get used to expressing in relatively high levels in cells. (C) On account that neurons are relatively stable cells and cell division rarely occurs, circRNAs theoretically accumulate during the process of development and aging.

### Functions

The superior abundance and stability in the mammalian brain prompt many researchers to explore the roles of circRNAs in the CNS. Nonetheless, the mechanisms deep into the details by which circRNAs take participated in have not been completely identified. As is well known, microRNA (miRNA), small single-stranded, and ncRNA, could downregulate post-transcriptional expression by binding 3′-untranslated region of target messenger RNA (mRNA) to adjust the progression of brain development and diseases (38). RNA-binding proteins (RBPs) acting as regulators participate in the generation, alternative splicing, transfer of RNAs, as well as post-transcripted regulation (39). Previous researches have revealed that circRNAs could not only serve as miRNA sponges to competitively inhibit the transcriptional regulation of miRNAs, but also alter mRNAs expression via RBPs (Figure 1B) (39, 40). Sometimes, a circRNA includes at least two miRNA binding sites (41). For example, circRNA CDR1as is predominantly expressed in excitatory neurons and owns more than 70 conserved binding sites, one of which candidate is miR-7. Cdr1as sponging to miR-7 could directly promote miRNA depletion. After vanishing Cdr1as locus, miR-7 was destabilized and neuronal activity was then changed, causing electrophysiological and motor disorders in mice (42). For another example, circDYM that possesses the binding site for miR-9 has been demonstrated to inhibit miRNA activity so that HSP90 ubiquitination would occur and consequently suppress the microglial activation (43). Therefore, we can speculate that the cirRNA-miRNA-mRNA axis is a promising direction in the CNS. Furthermore, circHomer1α, a neuronal-enriched circRNA, interacting with neuronal RBP HuD could adjust the expression of synapses. And circHomer1α disorder has been found in the patients with bipolar disorder and schizophrenia (44). Last but not least, certain circRNAs might directly perform as templates for translation (Figure 1B) (45, 46). An early study found that an endogenous circRNA called circPINTexon2 was used to encode a tumor-suppressive peptide during the proliferation of glioblastoma cells, suggesting the potential indicator of this circRNA as diagnostic biomarker for brain tumor (47). However, how circRNAs are excellently translated into functional proteins in normal physiological or pathological conditions need to further clarify.

## CircRNAs as Potential Biomarkers for Neurological Impairments and Diseases

Cerebrovascular autoregulation is extraordinarily important to maintain the physiological function of brain. Newborns, especially those premature neonates with higher susceptibility, have imperfect cerebrovascular structures and regulation mechanisms (48, 49). When asphyxia happens, the cerebrovascular self-regulation is gradually lost, leading to cerebral edema and even worse, cerebral hemorrhage. It is demonstrated that cerebral hypoperfusion could result in white matter damage (WMD), BBB damage, neuronal oxidative stress, and cell death (50). Fortunately, rat models of ischemia reperfusion injury have suggested that circRNAs were sensitive to cerebral ischemia (40). So, we are aware that circRNAs expression may have a significant influence on CNS. Moreover, the disappearance of rather rich circRNAs can also be applied to mark diseases (14). And there will be a potential chance in changing the level of dysregulated circRNAs to improve brain damage.

Due to tissue and cellular structure specificity of circRNAs, increasing studies have been revealed that circRNAs are connected to neurological dysfunctions caused by brain damage (13, 51). Oligodendrocytes (OLs) and myelin have been demonstrated to downregulate in the course of WMD, along with functional loss of synaptic transmission, eventually leading to white matter atrophy (52–54). In addition, synapses play a vital role in information transfer between neurons, and are crucial to the complex signal processing and network in CNS (55, 56). Circular RNAs multiply in abundance during fetal development and the formation of brain structure occurs before birth (37). These may be the main causes of long-term neurological deficits reflected in surviving children of brain damage. To sum up, circRNAs have the potential chance to serve as diagnostic and prognostic indicators.

### Neurodevelopmental Impairments

The early injury to the developing brain may cause circRNA disorder, recent, or long-term neurodevelopmental dysfunctions via diverse mechanisms. The circRNA Runx1t1, Pde5a, and Rtn4 three are differentially expressed in hippocampus and prefrontal cortex where learning and long-term memory take place, and their inhibition may perfect memory performance as well as cognitive function (57). Based on how circRNAs act in CNS, the relationship will be concluded between circRNAs and neurodevelopmental impairments in the aspects of intelligence, cognition and behavior, as well as others (Table 1).

**Table 1:** Overview of circRNAs in neurodevelopmental impairments.

**Neuro developmental impairments**	**Disease or injury model**	**Specific circRNAs**	**Corresponding miRNAs/RBPs**	**Possible biological process**	**Possible signaling pathway**	**References**
Intelligence	Human prenatal neocortex Long-term potentiation and long-term memory impairments in adult mice model	circSATB2	–	Neuronal development	BDNF signaling	Chen et al. (37), Jaitner et al. (58)
Cognition	BTBR mice model	circCdh9	–	Neuronal development and maintenance	Activity-dependent signaling pathways	Gasparini et al. (17)
	Genetic knockdown mice model	circHomer1α	HuD	Neuronal excitability and synaptic plasticity	–	Zimmermanet al. (44)
	Transgenic Alzheimer's disease mouse model Human blood samples	circHDAC9	miR-138	Synaptic plasticity	Sirt1 pathway	Lu et al. (59)
Behavior	Middle cerebral artery occlusion in mouse model	circTLK1	miR-335-3p	Neuronal injury and plasticity	The expression of TIPARP	Wu et al. (40)
Epilepsy	TLE blood samples	circ-EFCAB2	miR-485-5p	Synaptic plasticity	Calcium channel	Li et al. (60)
		circ-DROSHA	miR-1252-5p	Inhibition of neuron cdepolarization	The decrease of ATP1A2	
	TLE rat model	circ_Arhgap4	miR-6328	Synaptic plasticity	Dopamine receptor D2	Gomes-Duarte et al. (61)

*BDNF, brain-derived neurotrophic factor; Sirt1, sirtuin-1; TIPARP, 2,3,7,8-tetrachlorodibenzo-p-dioxin (TCDD)-inducible poly-ADP-ribose polymerase; TLE, temporal lobe epilepsy; ATP1A2, alpha 2 subunit of Na+, K+-ATPase*.

#### Intellectual Deficiency

Intellectual deficiency seriously affects the quality of life both of children themselves and their families. Circular RNA SATB2 from corpus callosum is significantly upregulated in the late stage of pregnancy (37). In a case report, the mutation of special AT-rich special sequence-binding protein 2 (SATB2) which encoded a DNA-binding protein can lead to severe intellectual disabilities in children, suggesting that SATB2 may play a disadvantageous role in nervous development (62). Furthermore, the relationship between SATB2 and learning and memory has been evidenced in mice experiments (58). And SATB2 directly activates transcription of forebrain embryonic zinc finger 2 and SRY-box 5 which are the essential genes for the development of subcerebral neurons (63). Collectively, more studies need performing to explain whether and how circSATB2 influences intellectuality.

#### Cognitive Impairment

The damage of cerebral gray and white matter has been confirmed to be closely related to cognitive abnormality in later life of preterm babies (64). Exon circCdh9, generated by Cdh9 gene, is validated to take part in autism spectrum disorder (ASD) (17). In other words, down-expressed circCdh9 may reflect cognitive problems. As we have mentioned before, circHomer1's original gene called HOMER1 was linked to psychiatric disorders (44). In addition, decreased levels of circHDAC9 in serum collected from cognitive impairment patients has been demonstrated, manifesting the connection between cognition and the steady-state levels of above transcripts (59).

#### Behavioral Disorder

Children with brain injury would exhibit abnormal behavior to some extent, such as attention deficit hyperactivity disorder (65). CircTLK1-knockout mice which lack certain circRNA expression in brain tissue, displayed reduced infarct volumes and neuronal damage, as well as significant improvements in neurobehavioral abnormalities. At the same time, circTLK1 sponging to miR-335-3p led to inhibited activity of miRNA, which aggravated subsequent neuronal injury (40). In consequence, the behavioral problems in children may be improved by regulation of related circRNAs expression.

#### Epilepsy

Epilepsy with the higher risk in infants is a chronic neurological disease, of which temporal lobe epilepsy (TLE) is the most common type of epilepsy. As its name suggests, TLE seizures begin in the temporal lobe cortex and then abnormal cortical signals are exhibited, characterized by hippocampal sclerosis, neuronal death, gliosis, and so on (66, 67). However, little is known between circRNAs and TLE in synaptogenesis, ion channels, and neuroinflammation (68). A previous study showed the analyses of circRNAs profiles in human TLE. Although circ-EFCAB2 was remarkably increased, circ-DROSHA was dramatically down-regulated confirmed in quantitative PCR (60). Circ-EFCAB2 can combine with miR-485-5p in order to regulate calcium channel. Circ-DROSHA binding to miR-1252-5p decreases the level of alpha 2 subunit of Na+, K+-ATPase (ATP1A2) which has been evidenced to participate in reducing the extracellular K+ and then inhibiting neuron depolarization (69, 70). Another experiment interestingly demonstrated that overexpressed circ-DROSHA weakened the neural damage of the TLE cell model (71). A recent study also confirmed the altered circRNAs expression during epileptogenesis (61). If circRNAs could impair paradoxical discharge, TLE would be suppressed.

### Neurological Diseases of Preterm Infants

**Figure 2:**
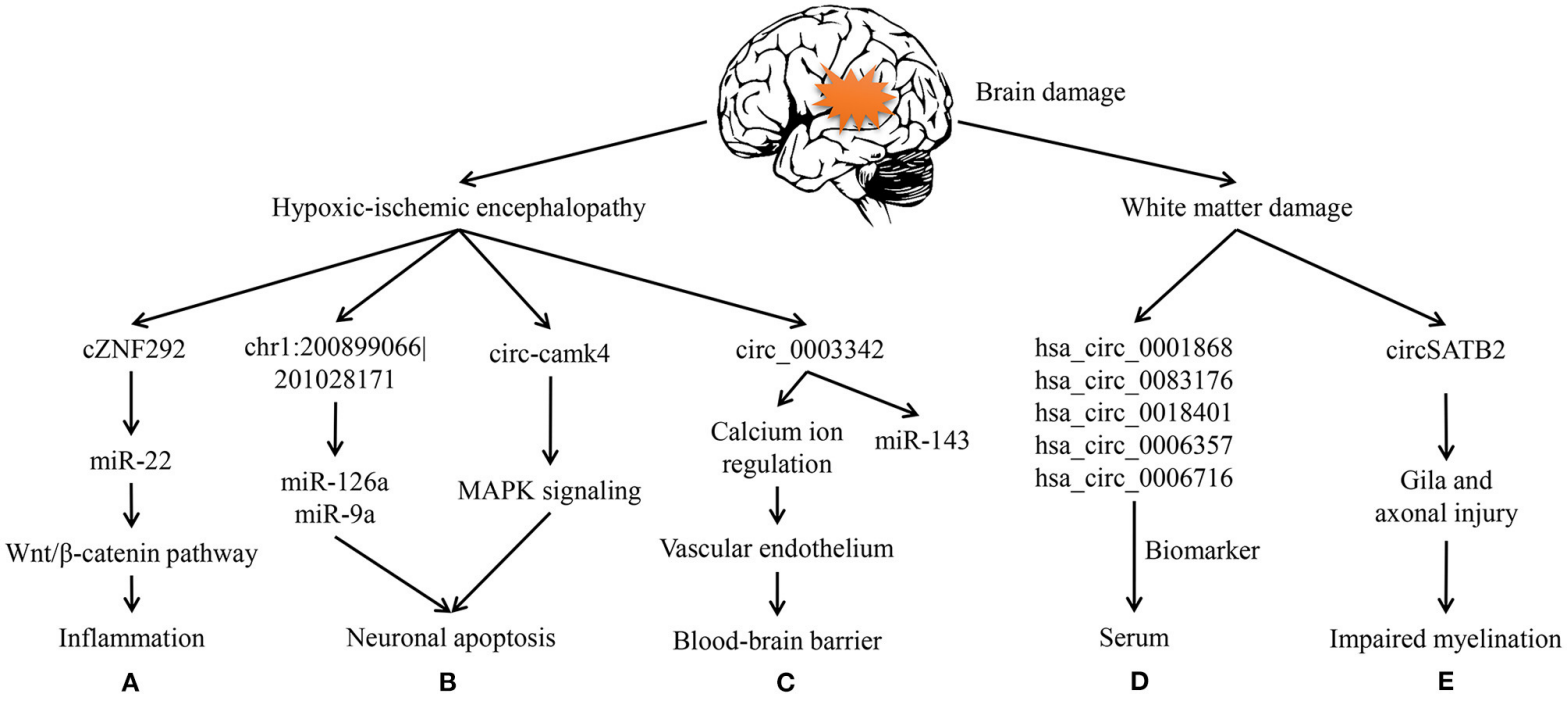
Circular RNAs in hypoxic-ischemic encephalopathy and white matter damage. **(A)** cZNF292 can act as a sponge of miR-22 and then participate in inflammation via wnt/β-catenin pathway of the oxygen-glucose deprivation/reperfusion (OGD/R) cell model. **(B)** In HIE rats, chr1: 200899066|201028171 can sponge miR-126a and miR-9a during apoptosis. circ-camk4 might participate in neuronal apoptosis by MAPK signaling pathway, which was showed through OGD/R neurons. **(C)** circ-003342 can sponge miR-143 to associate with vascular endothelium thereby regulate the blood-brain barrier in OGD/R brain microvascular endothelial cells. **(D)** hsa_circ_0001868, hsa_circ_0083176, hsa_circ_0018401, hsa_circ_0006357, and hsa_circ_0006716 are increased in premature infants serum to be biomarkers of white matter damage. **(E)** circSATB2 can regulate the extension of axons via animal and cell studies on schizophrenia.

The brain damage in early life does influence the prognosis of affected children. Hirosuke and his colleagues investigated 3-year-old children's neurodevelopmental outcomes who were born <500 g. Through follow-up visits and data analyses of these survivals, their group made a great discovery that cystic periventricular leukomalacia, a severe category of brain damage in premature infants, was the high risk factor for neurological consequences (adjusted RR:1.40; 95%CI:1.13–1.73; *P* < 0.01) (4). So we will first focus on hypoxic-ischemic encephalopathy (HIE), an important component of the brain damage. The WMD and its related neuronal malformations are then discussed (Figure 2).

#### Hypoxic-Ischemic Encephalopathy

Neonatal HIE refers to hypoxic-ischemic (HI) damage in brain caused by perinatal asphyxia and hypoxia, following a series of clinical neurological manifestations. What's worse, subsequent cascading reactions after cerebral ischemia-reperfusion may cause considerably severe brain damage (72). In other words, both cell death and apoptosis might to be caused by HI, which potentially leads to further secondary brain injury in adjacent tissues (73). In addition, such damages would alter the expression and release of circRNAs in cells. A current research has showed that circRNAs expression in neonatal rats following HI brain damage was greatly changed which included 20 upregulated and 46 downregulated circRNAs (74). In another mice model, circDLGAP4 overexpression significantly decreases infarct areas (75). Based on the above research results, circRNAs may serve as the potential role of novel treatment target in ischemic brain damage.

It is critical to appreciate that neuroinflammation is the main driver in injury to the developing brain and greatly amplified after HI insult. That is, inflammation inhibits conveying messages between neurons but increases the sensitivity of immature brains to hypoxic so that cerebral injury will be prolonged (76). What's more, oxidative stress is considered as the contributor to brain injury working in combination with inflammation (77). According to previous reports, microglia, astrocytes and endothelial cells activated by cerebral hypoperfusion can release reactive oxygen species (ROS) and toxicity of excitatory amino acids (78). And both excessive ROS and relative amino acids will further promote inflammatory cytokines and activate various cell signaling pathways, which in turn bring on immune responses, neuronal apoptosis and necrosis. This theory is widely applied in oxygen-glucose deprivation models (79). Through the neural stem cells experiment of oxygen-glucose deprivation/reperfusion (OGD/R)-induced injury, cZNF292 was over-expressed and then ORD/R injury exacerbated. Instead, cZNF292 had negative influence on miR-22, after which the insult was getting better by the way of wnt/β-catenin pathway (Figure 2A) (80). This signaling has been evidenced in importance for the differentiation of neurons and the formation of neural circuits, which are likely to be relevant to the arrangement of cerebrovascular and BBB (81). Therefore, this pathway will possibly have a huge impact on young brains.

Nevertheless, apoptosis is essential for the development of immature CNS. When the energy supply is significantly reduced, apoptotic cell death will occur, and at the same time, cell neurosis would take place in rather severe cases (49). Lin et al. made use of OGD/R model to analyze circRNAs in mouse hippocampal HT22 cells, and then speculated that several circRNAs may be involved in apoptosis, metabolism, and immune, which pathways have been widely studied in HIE (82). chr1: 200899066–201028171 can, respectively, sponge miR-126a and miR-9a to involve in hypoxia-induced neuronal apoptosis (Figure 2B) (11, 74). For another example, circ-camk4 could promote neuronal apoptosis, and might to act in MAPK signaling pathway which is related to the pathogenesis of cerebral ischemia reperfusion injury (Figure 2B) (83). Thus, apoptosis is regarded as a vital component of the neuronal cell death in neonatal HIE.

Blood-brain barrier selectively allows molecules to cross the barrier in order to protect the neonatal brains in physiological condition (84). Therefore, BBB damage is not only a consequence of but also a contributor to the progression of HI. Besides, cerebral microvascular endothelial cell dysfunction caused by cerebral ischemia-reperfusion is the initial phase of BBB destruction. It has been evidenced that certain circRNAs could improve vascular endothelial dysfunction (85). Modulation of circRNAs related to endothelial cell damage is beneficial to preserve the BBB integrity. For example, Liu et al. suggested that circRNAs may regulate calcium ion and facilitate signal transduction so as to modulate the function of vascular endothelium avoid cell swelling, and lysis (Figure 2C) (84). So, BBB protection has been regarded as a potential therapeutic strategy for ischemic encephalopathy. The molecular and cellular mechanism of HIE in BBB dysfunction remains to be elucidated. Similarly, the role of circRNAs in the pathogenesis of HIE is largely unknown. Consequently, further researches are needed to be done around circRNAs and BBB protection as well as inflammation in the developing brain.

#### White Matter Damage

White matter damage in premature infants is the predominant class of neonatal brain damage (86). It features high risk of disability, leading to severe impairments of neurological functions, such as cerebral palsy, cognitive impairment, epilepsy, and so on (52, 87). But even worse, for lack of specific clinical characteristics, its clinical diagnosis is largely depended on neuroimaging techniques which are called retrospective analyses. Cranial ultrasound (cUS) and MRI are the main methods for clinical diagnosis (88, 89). On the one hand, cUS is more convenient for the reason that it can be conducted in the ward. On the other hand, it's more suitable for cystic lesions and bigger focus while the vast majority of WMD is the diffuse type. As is known to all, MRI is not only used to analyze lesions' size, range, and location, but also as the gold standard of this disease (86). Nevertheless, it is the sedation before and during examination, and the movement to imaging department, as well as too much time spent on scanning, that make it more or less difficult to complete MRI, which very much result in delayed diagnosis for some severe patients.

Taking all the facts into consideration, there is an urgent need for early detection markers and novel therapeutic targets of WMD in preterm infants. Luckily, our team previously analyzed the whole blood samples of preterm infants who have had WMD and then identified that the expression profiles of circRNAs had greatly changed following brain injury (Figure 2D) (90). In hence, we suppose that circRNAs may act as an important regulator in the pathological changes and dysfunction of CNS.

At the same time, the white matter is featured in myelin, a critical component produced by OLs that facilitate the efficient transmission of electrical signals and then enable communication between gray matter neurons throughout various areas (91). Moreover, myelin can rapidly transfer action potential along axons, and myelination promoting synapses has been evidenced (92). As main cells in white matter between the GA of 24–30 weeks, premyelinating oligodendrocytes (pre-OLs) can be differentiated into mature OLs mainly after 32 weeks of gestation, which partially explains the highest incidence of WMD during this period (93). So selective pre-OL death seems to be the predominate process in diffuse WMD, eventually causing the failure of myelination in the developing white matter. Thus, pre-OL injury could also give rise to dysfunction of axonal development and ultimately axonal degeneration (65). Therefore, stimulating myelination and accelerating the recovery of white matter is possibly thought to account for effective treatments for WMD. However, little is known about circRNA function in pre-OL injury and repair during and after WMD. For this reason, whether circRNAs contribute to pre-OL damage in the course of WMD remains poorly understood.

Typically, loss of glia and axons has been acknowledged for many years to be the characteristic of cystic WMD (3). For preterm infants, the active axonal development in cerebral white matter could make nerve fibers particularly vulnerable. In CNS, the majority of axons cannot regenerate after injury, which likely explains irreversible neurological dysfunctions caused by CNS trauma (94). In addition to promote neuronal development, SATB2 regulates the extension of axons throughout the corpus callosum to participate in synaptic transmission (Figure 2E) (95).

Current researches of WMD have rarely involved the role of pro-inflammatory and anti-inflammatory in brain repair. Microglia, accounting for 10–20% of all glial cells, are the innate immune cells and the main regulator cells for the repair/regeneration of the CNS (96). Excessively activated microglia in white matter can destroy the axons and myelin sheaths of neurons, and ultimately lead to the declines in cognitive, motor, and other functions in various degrees. In other CNS diseases, circRNAs could regulate the progression through neuroinflammation. For instance, circHivep2 was evidenced in promoting microglia activation and the release of inflammatory factors via miR-181a-5p/SOCS2 pathway (97). Over-activated microglia may be harmful to axons and myelin in return. So, excessive inflammation could affect neuron development and inflammation is considered as the main cause in diffuse white matter lesions. If circRNAs could impair immoderate inflammation, WMI would be repressed.

## Conclusion and Future Perspective

The above reviewed findings emphasize the relevance of circRNAs as bioactive molecules regulating the neurodevelopmental conditions. The overall conclusions from the works presented here are: (A) Premature birth plays a major and early role in neurodevelopmental diseases; (B) Regulatory circRNAs expression and activity dynamics regulate, directly and/or indirectly, gene expression networks involved in brain damage; (C) CircRNAs have a direct impact on neurodevelopment and brain injury.

The detailed biogenesis of circRNAs remains complicated. The molecular and physiological functions of most circRNAs are still unknown, but there is a tendency that the focus in this area would generate tremendous growth due to the progressed methods of overexpression, knocking out, and knocking down in specific circRNAs. At present, researches are mostly focused on cerebroma and stroke in adults. Circular RNAs wrapped by exosomes or cell vesicles could pass through the incomplete BBB resulted from brain injury, so that circRNAs are available in peripheral blood. Circular RNAs have emerged as the new type of molecules with intriguing molecular functions and potential biomarkers of related diseases. For example, due to elevated levels in plasma, circPDS5B, and circCDC14A could be potentially valuable biomarkers for acute ischemic stroke and target for therapeutics in stroke outcomes. Overexpressed circ-CDC45 has been confirmed to be active in the progression of glioma proliferation and invasion, and predict its adverse prognosis (98). Especially, the BBB of premature infants with high permeability is imperfect, making circRNAs possible to be biomarkers of WMD.

In addition, the functional interaction between different classes of ncRNAs, such as circRNAs and corresponding miRNAs and RBPs, provides an additional layer of complexity, in which multiple circRNA-signaling pathways orchestrate the response to the cerebral injury. However, most of previous studies focused on a particular circRNA while ignore that various molecules are functionally altered in the CNS diseases. So, the diversity raises the challenge of aiming the regulation of circRNAs to better protect from brain damage. The cirRNA-miRNA-mRNA code is the focus of nowadays researches. Nevertheless, we can speculate that additional RNA classes present in the CNS can be affected by and associated to neurodevelopment, including highly abundant miRNAs.

The increasing roles of circRNAs in neurological diseases also drew our attention. Five circRNAs were found differentially expressed through RNA high-throughput sequencing (90). Preliminary work has shown that these circRNAs were highly correlated with WMD of preterm infants, and had better stability. In order to explore whether circRNAs can serve as WMD biomarkers, we plan to investigate the expression of certain circRNAs in peripheral blood and then analyze their effects by taking imaging examinations as the gold standard.

In spite of numerous studies in circRNAs, there remains a few researches in diseases associated with premature infants. All these results mentioned in this article demonstrate the unique prospect of circRNAs as clinical diagnosis markers and therapeutic targets, and neuroprotective agents for brain damage of preterm infants, but still need confirming in further continuous studies by the use of large quantities of animals, clinical samples and even patients. Early neurological interventions are closely related to the patient's quality of the rest of life. So, the application of circRNAs as neuro-biomarkers can be exploited in the near future, which could unbelievably make an increase in the possibility for premature infants to live better in the world.

## Author Contributions

QG was the primary author of the review. HL and JM assisted with writing and provided edits. All authors approved this version.

## Funding

This work was supported in part by the Postgraduate Research and Practice Innovation Program of Jiangsu Province (SJCX20_0057) and in part by the Fundamental Research Funds for the Central Universities (3224002112D).

## Conflict of Interest

The authors declare that the research was conducted in the absence of any commercial or financial relationships that could be construed as a potential conflict of interest.

## Publisher's Note

All claims expressed in this article are solely those of the authors and do not necessarily represent those of their affiliated organizations, or those of the publisher, the editors and the reviewers. Any product that may be evaluated in this article, or claim that may be made by its manufacturer, is not guaranteed or endorsed by the publisher.
